# The nuclear dynamic of CDC48 is affected during the immune response in plants

**DOI:** 10.1080/15592324.2025.2488104

**Published:** 2025-04-01

**Authors:** Damien Inès, Aymeric Leray, Pascale Winckler, Pierre-Emmanuel Courty, David Wendehenne, Claire Rosnoblet

**Affiliations:** a Université Bourgogne Europe, Institut Agro Dijon, INRAE, UMR Agroécologie, Dijon, France; b Laboratoire Interdisciplinaire Carnot de Bourgogne CNRS UMR 6303, Université Bourgogne Europe, Dijon, France; c Dimacell Imaging Facility, L’Institut Agro Dijon, INRAe, INSERM, Université Bourgogne Europe, Université Marie & Louis Pasteur, Dijon, France

**Keywords:** Cell division cycle 48, plant immunity, biophysics, fluorescence correlation spectroscopy, cryptogein

## Abstract

Plants are continuously challenged by a myriad of pathogenic microorganisms, including bacteria, viruses, fungi and oomycetes, against which they must defend themselves. The protein Cell Division Cycle 48 (CDC48), a key player of ubiquitin-proteasome system which segregates and remodels ubiquitinated proteins for degradation, is known to be mobilized during plant immunity. Moreover, the characterization of the nuclear role of CDC48 is of interest, in particular its regulation in nuclear processes such as chromatin remodeling, DNA repair and gene expression. In this regard, its nuclear functions during plant immunity remain underexplored. This study investigates the dynamics of CDC48 during plant immune responses. The biophysical analysis using the Fluorescence Correlation Spectroscopy (FCS) on tobacco leaves stably overexpressing GFP-CDC48 revealed that the nuclear dynamics of CDC48 changed after treatment with cryptogein, an elicitor of immune responses. FCS analysis revealed an increase of the nuclear mobility of CDC48 and a faster interaction of CDC48 with a wide range of nuclear partners shortly after cryptogein treatment. Overall, our study shows a nuclear regulation of the interaction of CDC48 with its potential partners and sheds new light on potential nuclear involvements of CDC48 following the triggering of defense mechanisms.

## Introduction

**Figure 1.**
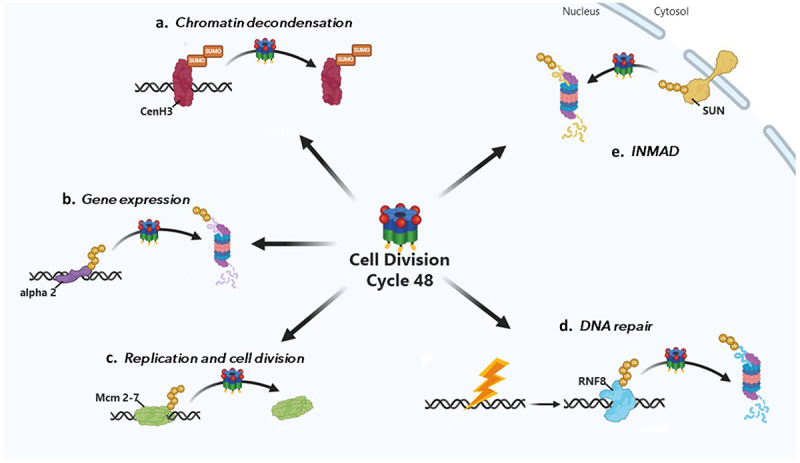
Nuclear functions of CDC48. (a) Chromatin Decondensation Regulation. In Arabidopsis, CDC48 interacts with sumoylated cenH3 to segregate it from chromatin, decondensing DNA regions required for ribosomal RNA synthesis, thereby promoting pollen tube growth.^11^ (b) Regulation of gene expression. CDC48 facilitates transcription by segregating the polyubiquitinated alpha2 transcriptional repressor and degrading it via the proteasome.^15^ (c) Replication and cell division regulation. CDC48 halts DNA replication by extracting ubiquitinated CMG helicase complexes through the recognition and segregation of the Mcm7 subunit.^9^ (d) DNA repair regulation. Following DNA damage repair, CDC48 segregates and targets to the proteasomal degradation the ubiquitin ligase RNF8.^13^ (e) INMAD. CDC48 plays a crucial role in the degradation of inner nuclear membrane proteins (INMAD), including SUN proteins, which are key regulators of nuclear morphology.^8^

In their environment, plants are exposed to various microorganisms, some of which being pathogenic (bacteria, viruses, fungi). As a result, they set up defensive reactions which are finely regulated.^1^ Studies have estimated that the expression of up to 20% of plant genes were significantly modified during plant immune responses.^1,2^ These regulated genes are involved in plant defense and encode for a myriad of proteins with various functions, including antimicrobial proteins and enzymes involved in the biosynthesis of metabolites.^1,3,4^ This large-scale and tightly transcriptional reprogramming can lead to a strengthening of the plant cell wall and, in some cases, trigger a localized cell death response (hypersensitive response) limiting the spread of pathogens.^4–6^ Several proteins related to the regulation of protein homeostasis, including Cell Division Cycle 48 (CDC48), are involved in plant immune response. CDC48 is an ATPase chaperone-like protein highly conserved protein across plants, yeast and mammals (known as Valosin Containing Protein, VCP, in mammals, or p97 in all organisms). CDC48 segregates ubiquitinated proteins from protein complexes and subcellular structures, such as membranes or chromatin, then remodels and sends them to the proteasome for degradation.^7^ Consequently, CDC48 is essential for protein quality control through the Ubiquitin Proteasome System (UPS). Additionally, it regulates diverse nuclear functions, such as cell cycle, DNA repair, chromatin remodeling, inner nuclear membrane protein associated degradation (INMAD) and gene expression^8–15^; Figure 1). In particular, CDC48 plays an essential role in the assembly and disassembly of protein – DNA complexes and in degradation of misfolded nuclear proteins.^16,17^ For example, CDC48 regulates transcription by segregating the polyubiquitinated transcriptional repressor alpha2 before sending it to degradation.^15^ More recently, CDC48 has been shown to regulate nuclear morphology through INMAD-mediated degradation of the SUN protein.^8^

CDC48 is known to be mobilized during plant immunity. For instance, CDC48 has been shown to specifically accumulate in cells in response to cryptogein, an elicitor produced by the oomycete *Phytophthora cryptogea* that triggers an immune response in tobacco. Moreover, cryptogein treatment in CDC48-overexpressing stable cell lines has been linked to premature HR-like cell death.^18,19^ CDC48 is also involved in the turnover of immune receptors and pathogenesis effectors.^20^ Furthermore, following tobacco mosaic virus infection, no necrosis was observed in leaves of tobacco plants overexpressing CDC48,^21^ highlighting its role in virus resistance. Nevertheless, while CDC48 is recognized as a new player in plant immune responses, its nuclear role has not yet been characterized. Our targeted study aims to characterize the nuclear dynamics of CDC48 during the plant immune response.

## Materials and methods

### Treatments and chemicals

*Nicotiana tabacum* cv. Xanthi plants stably overexpressing GFP-CDC48 (green fluorescent protein tag at the N-terminal part of CDC48,^18,21^ were grown at 24°C, 16 h day/8 h night. Cryptogein was purified according to Bonnet et al.^22^ and stored at −20°C as a 100 μM stock aqueous solution. Tobacco leaves were infiltrated either with water or with cryptogein (final concentration of 100 nM) using a syringe. All the experiments were performed on three independent biological replicates.

### Fluorescence correlation spectroscopy (FCS)

FCS analysis were performed on the fourth leaf of two-month-old tobacco plants overexpressing GFP-CDC48, 30 minutes after water or cryptogein infiltration. GFP-CDC48 overexpressors exhibit approximately twice the endogenous level of CDC48.^21^ FCS measurements were conducted on a Nikon A1-MP two photon scanning microscope (Nikon, Japan) with a × 60 Apo IR objective (NA: 1.27, Water Immersion, Nikon). A 880 nm laser (Chameleon Vision II, Coherent) provided the excitation light. Fluorescence emission of GFP was collected through a band-pass emission filter FF01–520/35 (Semrock), using a Single-Photon Avalanche Diode detector connected to a Picoquant single photon counting module (Picoquant, Germany). Measurements were performed at room temperature (22°C) on cell nucleus. Each FCS measurement was recorded during 120 s to obtain a sufficient ACF accuracy. Each point represents the mean of three measurements per nucleus. Fifteen nuclei were recorded per experimental condition. Fluorescence time traces were analyzed with EasyFCS, a home-made software (free of charge: https://github.com/ayleray/EasyFCS.) written in MATLAB (version R2023B; The MathWorks, Natick, MA) that calculates the autocorrelation function (ACF also noted G(τ)) with the time-tag-to-correlation algorithm developed by Wahl et al.^23^ EasyFCS was also used for filtering the ACF and reducing the auto-fluorescence (fluorescence lifetime) by separating the intrinsic fluorescence from the scattered light.^24^ A moving average detrending with a sliding window of 3 s was finally applied in EasyFCS to remove the slow intensity variations due to cell movements.

To estimate the association and dissociation rates at immobile binding sites within live cells, we have fitted these pre-processed ACFs with the full model for diffusion and binding developed by Michelman-Ribeiro et al.^25^:

$$G\left(\tau \right)=\frac{w_{0}^{2}z_{0}}{8N{\left(2\pi \right)}^{3/2}}\int \;\;\int \frac{\left(1-\phi \right)e^{-\frac{k_{1}\tau }{2}}+\left(1+\phi \right)e^{-\frac{k_{2}\tau }{2}}}{2}\text{\textbackslash{}break}\left(e^{-\frac{w_{0}^{2}}{4}q_{r}^{2}-\frac{z_{0}^{2}}{4}q_{z}^{2}}\right)2\pi q_{r}dq_{r}dq_{z}$$

Where *N* is the number of molecules in the observation volume, w_0_ and z_0_ are the lateral and axial extents of the Gaussian beam defined as the distances where the intensity is reduced by a factor 1/e^2^, and *q*_*r*_ and *q*_*z*_ are the lateral and axial Fourier transform variables, respectively.

*ϕ*, *k*_*1*_ and *k*_*2*_ values are given by:

$$\phi =\frac{{\left(k_{on}^{*}+k_{\mathrm{off}}\right)}^{2}+\left(k_{on}^{*}-k_{\mathrm{off}}\right)\left(q_{r}^{2}+q_{z}^{2}\right)D}{\left(k_{on}^{*}+k_{\mathrm{off}}\right)\sqrt{{\left(D\left(q_{r}^{2}+q_{z}^{2}\right)+k_{on}^{*}+k_{\mathrm{off}}\right)}^{2}-4D\left(q_{r}^{2}+q_{z}^{2}\right)k_{\mathrm{off}}}}$$

$$k_{1}=D\left(q_{r}^{2}+q_{z}^{2}\right)+k_{on}^{*}+k_{\mathrm{off}}+\sqrt{{\left(D\left(q_{r}^{2}+q_{z}^{2}\right)+k_{on}^{*}+k_{\mathrm{off}}\right)}^{2}-4D\left(q_{r}^{2}+q_{z}^{2}\right)k_{\mathrm{off}}}$$

$$k_{2}=D\left(q_{r}^{2}+q_{z}^{2}\right)+k_{on}^{*}+k_{\mathrm{off}}-\sqrt{{\left(D\left(q_{r}^{2}+q_{z}^{2}\right)+k_{on}^{*}+k_{\mathrm{off}}\right)}^{2}-4D\left(q_{r}^{2}+q_{z}^{2}\right)k_{\mathrm{off}}}$$

Where *D* is the diffusion coefficient and *k*_on_^*^ and *k*_*off*_ are the association and dissociation binding rates to an immobile substrate (in s^−1^).

We have then calculated four parameters: the number of molecules in the observation volume *N*, the diffusion coefficient *D*, and the association and dissociation rates (*k*_on_^*^ and *k*_*off*_). We used the trust-region-reflective least squares algorithm to minimize the sum of the squared error between the experimental data and the full model. To retrieve the value of the association rate *k*_*on*_ in M^−1^s^−1^, we have divided k_on_* by the concentration c:

$$k_{\mathrm{on}}=\frac{k_{on}^{*}}{c}=k_{on}^{*}\frac{\pi^{3/2}N_{A}w_{0}^{2}z_{0}}{2^{3/2}N}$$

With *N*_*A*_, the Avogadro constant. The dimensions of the observation volume (w_0_ and z_0_) were fixed to w_0_ = 0.22 μm and z_0_ = 0.6 μm after calibration with a 10 nM concentration solution of Atto 488 in water whose diffusion coefficient at 37° is 540 μm^2^/s (ref: Peter Kapusta, PicoQuant GmbH Application Note, 2010 https://www.picoquant.com/images/uploads/page/files/7353/appnote_diffusioncoefficients.pdf).

## Results

A biophysical analysis was performed using Fluorescence Correlation Spectroscopy (FCS) to characterize the nuclear dynamics of CDC48 during the early events of the plant immune response triggered by cryptogein. FCS analyses were performed on tobacco leaves stably expressing GFP-CDC48, 30 minutes after water or cryptogein infiltration. The association/dissociation (Kon/Koff) model was chosen to provide insights into CDC48 nuclear dynamics through three parameters: the diffusion coefficient (D, in µm^2^ s^−1^) is related to the mobility of CDC48 across a defined area; the rate of association (Kon, in M^−1^ s^−1^) reflects the interaction binding speed and the number of CDC48 partners; the dissociation rate (Koff, in s^−1^) among CDC48 and its partners. We also calculated the dissociation constant Kd = Koff/Kon (in M) to characterize the binding affinity. FCS is a reliable and efficient technique for determining the cellular dynamics of a protein.^26^ This technique has already been used in plants to study the dynamics of membrane proteins within the cell.^27–29^

**Figure 2.**
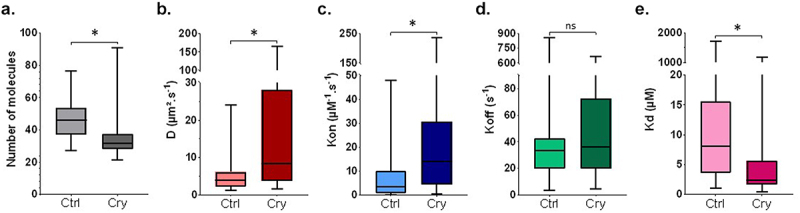
Biophysical analysis of CDC48 nuclear dynamics, using the FCS technique, 30 minutes after the treatment with cryptogein. (a) Number of molecules of GFP-CDC48 within the analyzed volume. (b) Diffusion coefficient of CDC48 (D, µm^2^.s^−1^). (c) Association rate of CDC48 with its partners (Kon, µM^−1^.s^−1^). (d) Dissociation rate of CDC48 with its partners (Koff, en s^−1^). (e) Dissociation constant of CDC48 with its partners (Kd, µM). All experiments were performed on leaf discs of tobacco plants stably overexpressing GFP-CDC48. Results are means of data from three independent biological replicates (± SD), n = 33 for the control condition and n = 30 for the condition treated with cryptogein for 30 min. (t-test, *p ≤ 0.005). Cry: cryptogein; n: number of measurements performed.

The number of GFP-CDC48 molecules was significantly reduced within the studied nuclear volume, from about 48 to 32 molecules, when comparing leaves infiltrated with water and with cryptogein (Figure 2a). The diffusion coefficient (D) and the association rate (Kon) of CDC48 in tobacco leaves significantly increased after cryptogein infiltration, from 3.5 µm^2^.s^−1^ to 8.4 µm^2^.s^−1^ and from 3.5 µM^−1^.s^−1^ to 14.1 µM^−1^.s^−1^, respectively (Figure 2b and c). In contrast, the dissociation rate (Koff) remains constant in leaves infiltrated with water or with cryptogein, of about 35 s^−1^ (Figure 2d). The kd value significantly decreased under cryptogein treatment, from 8,1 to 2,4 µM, suggesting an increase of the binding affinity of CDC48 for its partners (Figure 2e).

## Discussion

**Figure 3.**
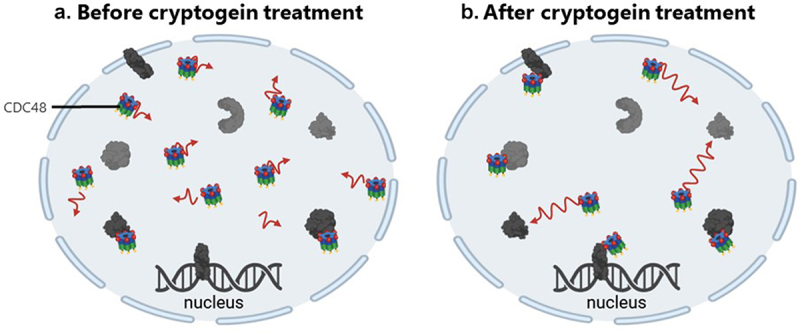
Schematic representation of the nuclear dynamics of CDC48 before and after cryptogein treatment. (a) Before treatment with cryptogein, nuclear CDC48 exhibits slow diffusion within the nuclei and interacts with a limited set of nuclear partners in diverse nuclear processes. (b) After cryptogein treatment, the nuclear concentration of CDC48 decreased and its diffusion increased. Additionally, CDC48 interacts more rapidly or with more nuclear partners. This enhanced mobility and interaction of CDC48 potentially contributes to the expression of defense-related genes or the maintenance of nuclear protein homeostasis. CDC48 protein partners are represented in dark gray. The length of the red arrow represents CDC48 mobility.

In our study, the diffusion coefficient (D) of CDC48 in tobacco leaves infiltrated with water was about 3.5 µm^2^.s^−1^. Our results are similar to those of Aker et al.^27^ reporting a FCS analysis of CDC48, suggesting an accurate calibration of the measured parameters. D increased significantly in tobacco leaves infiltrated with cryptogein to 8.4 µm^2^.s^−1^, suggesting that free CDC48 (not bound to partners) is more mobile within the nucleus in the early stages of the immune response. At the same time, we measured a decrease of the number of GFP-CDC48 molecules within the studied nuclear volumes, indicating that the cryptogein treatment leads to a reduction of the number of CDC48 molecules in the nucleus. Additionally, the association rate (Kon) significantly increased after cryptogein elicitation, suggesting that CDC48 interacts more rapidly and/or with more nuclear partners. However, the dissociation rate (Koff) remained constant in tobacco leaves regardless the treatment. So, we assume that as long as CDC48 binds to a nuclear partner, the induction of the immune response does not modify the length of the interaction with its partners. Given the increased Kon and unchanged Koff, the Kd value decreased significantly. Therefore, the affinity of CDC48 for its nuclear partners is higher after the triggering of plant defense mechanisms. CDC48 is involved in various nuclear processes, including chromatin remodeling, gene expression and INMAD.^8,11,12^ During the activation of plant defense mechanisms, a large-scale transcriptional reprogramming occurs within the nucleus.^1^ In our study, after cryptogein infiltration, we measured an increase of the diffusion coefficient (D) of CDC48 in the nucleus, suggesting its nuclear remobilization after the activation of the plant immune response. Furthermore, the increase of the association rate (Kon) suggested that CDC48 interacts with a broader and novel set of nuclear partners, potentially contributing to the expression of defense-related genes and the maintenance of nuclear protein homeostasis, as described in processes like chromatin-associated degradation (CAD) and INMAD. The decrease of the dissociation constant (Kd) further supports that CDC48 is specifically remobilized during plant immunity and strongly engaged with its new nuclear partners. These findings provide new insights into the nuclear role of CDC48 during defense mechanisms, suggesting that CDC48 is a nuclear regulator of plant immunity (Figure 3).

## Data Availability

Data are either provided in the manuscript in the main document or as supporting information or can be requested from the corresponding author.
